# Nottingham Hip Fracture Score Versus Surgical Outcome Risk Tool in Predicting 30-Day Mortality in Hip Fracture Patients in a District General Hospital in the United Kingdom

**DOI:** 10.7759/cureus.100380

**Published:** 2025-12-29

**Authors:** Surya Prasad, Babajide Obidigbo

**Affiliations:** 1 Orthopaedic Surgery, York Hospital, York, GBR; 2 Trauma and Orthopaedics, London North West University Healthcare NHS Trust, London, GBR

**Keywords:** compare, hip fracture, mortality score, nhfs, sort

## Abstract

Introduction and aims

Neck of femur fractures (NOFFs) represent high costs to the National Health Service and the elderly population’s quality of life. It is important to recognize vulnerable patients for early optimisation. This is a retrospective study of 157 NOFF patients to assess the 30-day, 60-day, six-month, and one-year mortality rates. The actual 30-day mortality was compared against the Nottingham Hip Fracture Score (NHFS) predicted 30-day mortality and the Surgical Outcome Risk Tool (SORT) version 2.0 30-day mortality.

Methods

This is a retrospective study conducted at York District General Hospital in the United Kingdom. The computerised patient data (CPD) records were accessed to find NOFF patients between 1/1/24 and 1/9/24 over a period of eight months. This allowed us to assess the 30-day, 60-day, six-month, and 12-month mortality rates. The NHFS and SORT version 2.0 (henceforth referred to as SORT) was calculated using the pre-defined parameters. The actual 30-day mortality was compared against the NHFS and SORT score, using the Z-test of proportions and the receiver operating characteristic to assess the model fit and statistical significance, with a p-value of 0.05 being the determining value. The Hosmer-Lemeshow (H-L) test was used to assess the goodness of fit for logistic regression models.

Results

The average age of the 157 patients was 83 years ±1.304 (±1.6%) (81.696-84.304; 95% CI intervals). The cohort was made up of 106 women and 51 men (106/157 women to 51/157 men). The mean NHFS was 5.0 - representing a 30-day mortality risk prediction of 5.99%. The mean SORT 30-day mortality was 8.28%. When compared to the actual 30-day mortality of 6.67%, the NHFS was not significantly different to this value (5.99% vs. 6.67%; p = 0.453; p > 0.05); however the SORT 30-day prediction of 8.28% was significantly higher than the actual mortality rate (8.28% vs. 5.99%; p < 0.00001); therefore, NHFS was significantly more accurate than the SORT score in predicting 30-day mortality for this particular cohort over this particular time period.

Conclusion

This paper suggests there is perhaps greater utility in predicting 30-day mortality through the use of the NHFS over the SORT tool; however, one must be cautious in determining clear superiority. The main rationale for this finding is that a dedicated tool, which incorporates measures relevant to the cohort most affected. Much work is yet to be done in fine-tuning predictor tools.

## Introduction

Neck of femur fractures (NOFFs) represent one of the highest costs to the National Health Service (NHS) and the elderly population's quality of life. NOFFs constitute a host of subsequent pathologies to the patient, including hospital-acquired infections, further falls and fractures, decreased independence and mobility, wound breakdown, poor nutritional intake, and increased frailty [1]. It is also important to consider the financial burden of NOFFs; Leal et al. conducted a retrospective study of over 30,000 NOFF patients [2]. The mean one- and two-year hospital costs after index hip fracture were £14,163 and £16,302, respectively [3]. The main predictors of one-year hospital costs were repeated hip fractures after discharge, likely secondary to poor mobility and increased instability. Other causes requiring hospitalisation included hip fracture-related complications, such as wound breakdown, hip dislocations, and prosthetic joint infections [3]. Total United Kingdom (UK) annual hospital costs associated with incident hip fractures were estimated at £1.1 billion [3]. It would be prudent, therefore, to develop a system to allow detection of vulnerable patients to allow us to pre-emptively optimise them, to allow decreased length of stay (LOS) and increased independence postoperatively.

Primary objective

The reasons for performing this study are multifactorial. NOFFs represent a large burden in the UK, with more than 70,000 cases reported annually [4]. Reported mortality rates are approximately 10% within 30 days and can reach up to 30% within one year [5]. This is a retrospective study of 157 NOFF patients who were admitted to a District General Hospital in the UK, with the primary aim being to assess actual 30-day mortality against the Nottingham Hip Fracture Score (NHFS) predicted 30-day mortality and the Surgical Outcome Risk Tool (SORT) version 2.0 30-day mortality.

Secondary objective

The secondary objective was to examine 60-day, six-month, and one-year mortality rates and contrast these rates with existing literature to assess the rates within this District General Hospital. This paper also examines each variable presented in the NHFS and SORT individually and corroborates and suggests a theory, with evidence from existing literature, as to why each variable contributes to patient mortality rates.

Tertiary objective

The tertiary and final objective of this paper is to assess the quantity and quality of information gathered during the admission process of patients and put forth suggestions on how they can be improved for this particular centre.

With the increasing age of the population and increased rates of osteoporosis, frailty, and comorbidities associated with poor outcomes, such as diabetes, cardiovascular dysfunction [6], it has become increasingly important to be able to recognise patients with high mortality risk scores. The use of mortality scores in this particular hospital was not routinely employed, and as part of a secondary aim, this paper wishes to promote the use of risk stratification scores as part of the initial clerking for patients. Several prognostic tools have been evaluated to support perioperative care, including the American Society of Anaesthesiologists (ASA) score [7], one of the most widely used systems. However, no single tool has demonstrated clear superiority in predicting perioperative risk among older patients.

However, to use risk stratification scores, we need to deem them accurate. The NHFS is more widely used than the SORT tool as a more specific score for NOFFs. This not only aims to assess the validity of these scores against one another, but also across the range of scores within the NHFS itself. In terms of future studies, if we can deem the NHFS or the SORT to be useful tools, they can be employed in the same hospital over a period of 12 months; the mortality rates and LOS could then be repeated to assess whether the use of early detection scores does offer a benefit, for patient welfare and financially for the trust.

## Materials and methods

This is a retrospective study conducted at York District General Hospital in the UK. The study’s primary objective is to analyse whether the NHFS or the SORT can predictably measure 30-day mortality of hip fracture patients. The computerised patient data (CPD) records were accessed to list the number of patients coded as 'Proximal Femur Fracture' and 'Neck of Femur Fracture’ between 1/1/24 and 1/9/24 over a period of eight months in this one particular district general hospital in the UK.

To obtain the secondary objective of this paper, CPD also allowed us to assess 30-day, 60-day, six-month, and 12-month mortality rates up until 1/5/25. Initially, 263 patients were included in the study. However, 24 patients who were wrongly coded as NOFFs but indeed had periprosthetic fractures and femoral shaft or distal femur fractures had to be excluded from the study. There were four patients who were too unwell to undergo any surgical fixation or had passed away before operative management were excluded from the study.

There were 78 further patients with NOFFs who had to be excluded from the study due to insufficient data points to calculate the NHFS and SORT; 78 patients had to be excluded due to missing Abbreviated Mental Test Score (AMTS). There were attempts made to minimise the missing data. Not only was CPD utilised to ascertain information regarding the variables needed to calculate each respective score, but we also utilised paper documents and looked through patients' Summary Care Records from their general practitioner. As a tertiary objective, the 78 patients with missing AMTS were looked into further to determine who had admitted the patient and whether any rationale for not completing the scoring was documented.

**Figure 1:**
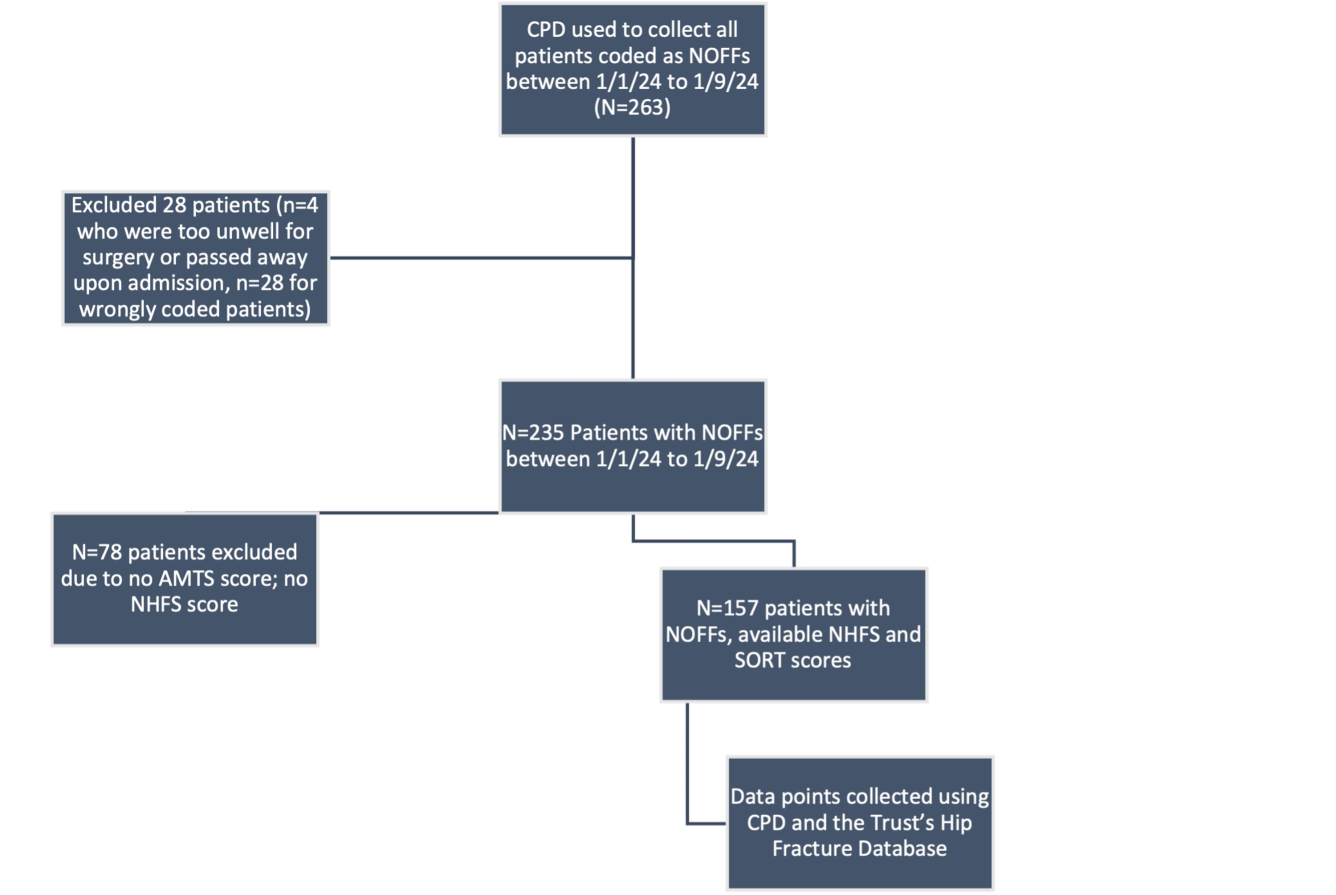
Initial patients included (n = 263) and the selection process down to the total number used in this paper (N = 157)

**Figure 2:**
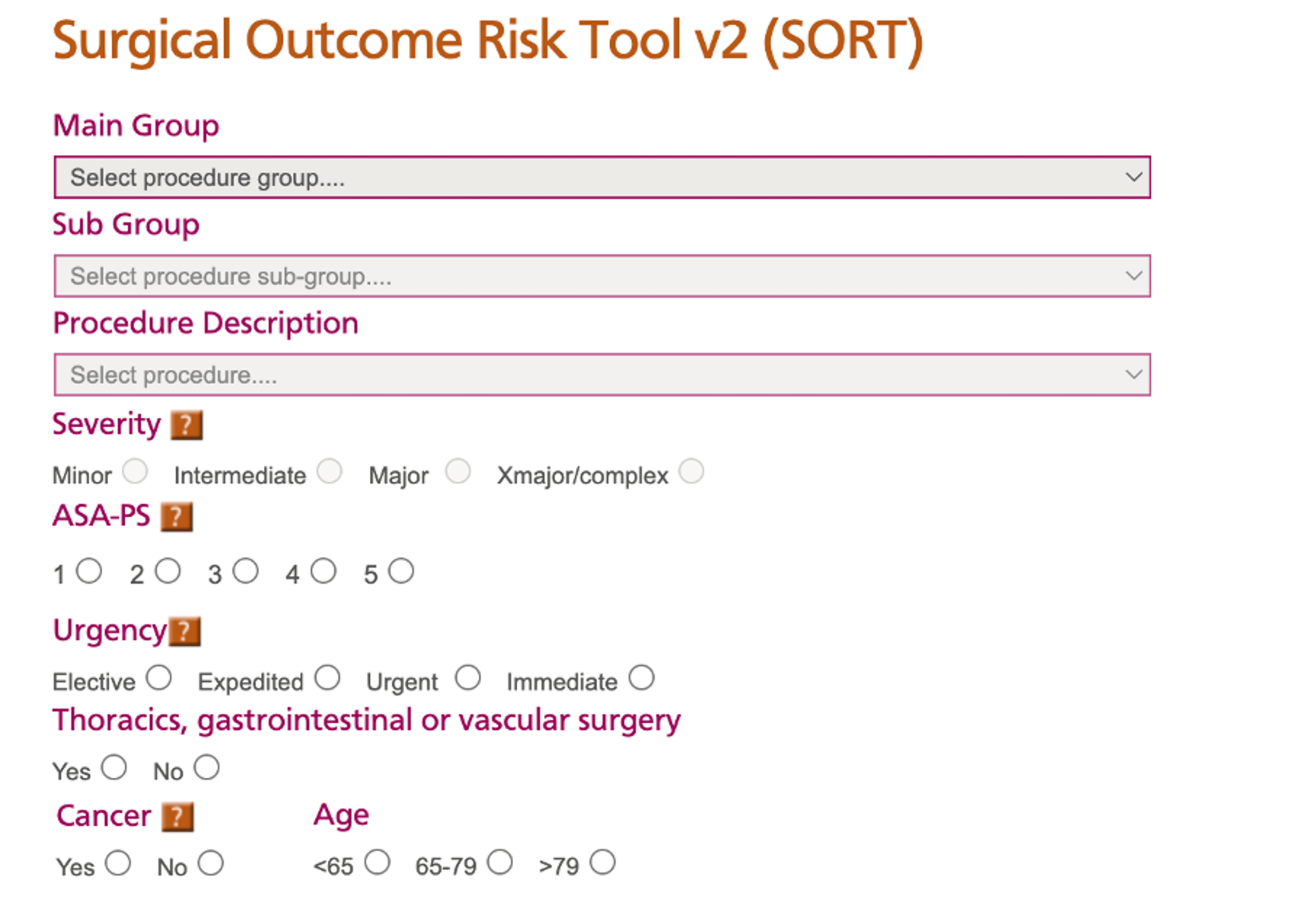
Surgical Outcome Risk Tool (SORT) scoring system used in this study The variables used in the SORT include the presenting diagnosis, the surgical procedure, the severity of the procedure, the ASA grade of the patient, the urgency of the planned surgical procedure, whether there is any thoracic, gastrointestinal, or vascular surgery planned, the presence of malignancy, and patient age.

**Figure 3:**
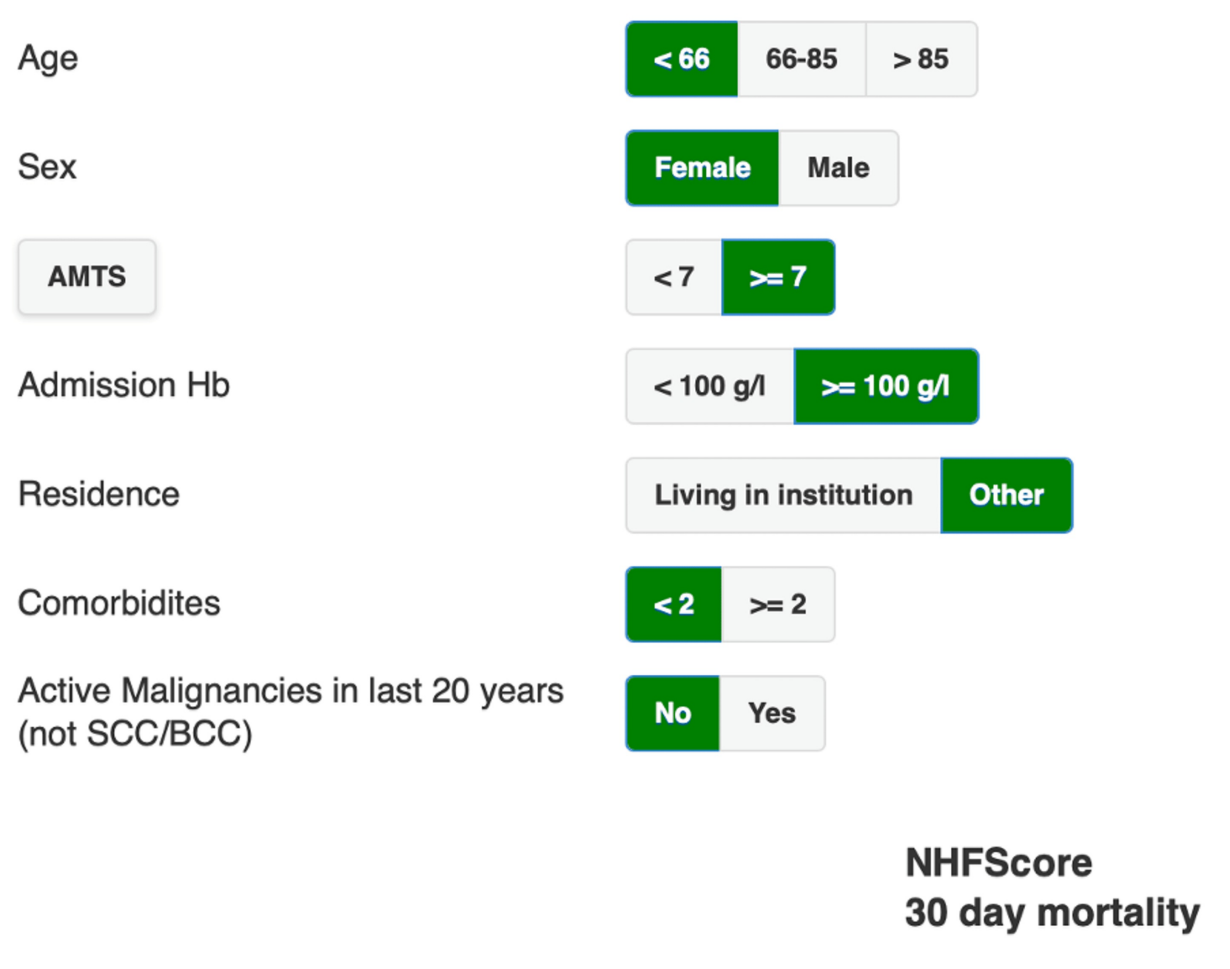
Nottingham Hip Fracture Score (NHFS) calculator utilised in this paper The variables include the age of the patient, their sex, the Abbreviated Mental Test Score (AMTS), haemoglobin (Hb) on admission (preoperative), their place of residence, number of comorbidities, and the presence of non-dermatological malignancies.

Figure 1 shows the study design pathway for including and excluding patients. A total of 157 patients with NOFFs were included in this paper. The inclusion criteria are specifically patients who had either an intracapsular or extracapsular NOFF suffered between 1/1/24 and 1/9/24 with sufficient documented information to calculate both the NHFS and the SORT score, the parameters of which are outlined in Figures 2-3. If relevant, the date of death would need to be recorded to assess the mortality rate for the cohort. All 157 patients included in the study were well enough to undergo operative fixation, either with a hip hemiarthroplasty (HH), intramedullary nail (IMN), or a dynamic hip screw (DHS).

The Information Technology Department stores data for all hip fracture patients as part of the National Hip Fracture Database. This was accessed to ascertain information regarding the patients’ demographics, preoperative mobility status, comorbidities, Best Practice Time (BPT), preoperative AMTS, operation date, ASA grade, and residential status. Figures 2-3 show the methods used to calculate the SORT and NHFS, respectively. The NHFS was calculated utilising the patients’ age, sex, pre-admission haemoglobin (Hb), preoperative AMTS, number of comorbidities, residential status, and whether there is a history of malignancy. The cutoffs for each variable are clear. For AMTS, the score was 7 or more to score 0; the cutoff for Hb was 100 g/dL or more to score 0. In terms of residential status, if the patient lived in any form of care home, assisted living with carers, or anything that was not their own home, independently without carers, they would score for this section. Comorbidities included pre-existing cardiovascular conditions (hypertension, atrial fibrillation, valvular disease, heart failure, or ischemic heart disease), cerebrovascular disease (stroke or transient ischemic attack), respiratory disorders (asthma or chronic obstructive pulmonary disease), malignant non-invasive skin cancer, renal disease, diabetes, or chronic gastrointestinal pathologies.

This information was gathered using the National Hip Fracture Database and CPD to access blood results and date of death, if relevant. The SORT version 2.0 (henceforth referred to as SORT) was utilised, which required the patients’ operative procedure, the ASA grade, the presence of malignancy, and age. Both scores were collected for all 157 patients. With regard to the NHFS, the mortality rate across the range of scores was analysed, and the Z-test of proportions was used to determine whether the predicted 30-day mortality (NHFS) was significantly different to the actual 30-day mortality. The actual 30-day mortality was compared against the NHFS and SORT scores, using a Z-test of proportions to assess the statistical significance of the difference. The data for six-month and 12-month mortality were also collected to be compared against the statistics quoted in relevant literature to allow comparison of this particular centre against longer-term predicted mortality rates. To achieve a standard error of 0.02 (or 2%) with a 95% CI +/- 4% would require 149 patients; therefore, we have a sufficient sample size of 157 patients to power this study. The Hosmer-Lemeshow (H-L) test was used to assess the goodness of fit for logistic regression models by comparing observed and expected event rates. All analyses considered a significance level of 5% (p-value < 0.05). The HL test is a statistical goodness-of-fit test used primarily to evaluate logistic regression models; it was used because logistic regression evaluates the calibration of the model-whether predicted risks align with actual event rates, which suits this study's objectives. The ROC was plotted to assess the sensitivity (true-positive rate) and specificity (false-positive rate) change across all possible thresholds, and DeLong's test for two independent ROC curves was utilised to assess whether there was a significant difference between the area under the curve (AUC) for NHFS versus SORT.

## Results

The average age of the 157 patients was 83 years ±1.304 (±1.6%) (81.696-84.304; 95% CI intervals). The cohort was made up of 106 women and 51 men (106/157 women to 51/157 men). This was a statistically significant difference in sex differences within the cohort (67.52% women vs. 32.48% men; p < 0.00001). Although there was a higher 30-day mortality in men compared to women (7.84% vs. 5.66%), this was not statistically significant (p > 0.05). Table 1 shows the demographics of the cohort with regard to sex and age ranges.

**Table 1:** Demographic distribution of the patients across the cohort (total N = 157)

Demographics	Number (N = 157)	Percentage (%)	30-day mortality (%)
Male	51	32.48	7.84
Female	106	67.52	5.66
Age ranges <66	6	3.82	0
Age range 66-85	80	50.96	1.25
Age range >85	71	45.22	12.68

Forty-one out of 157 (26.11%) had an AMTS of under 7, and 116/157 (73.89%) patients had a score of 7 or more on the AMTS. Twelve out of 41 (25.53%) of those with AMTS less than 7 died within 12 months, compared to 20/116 (17.24%) with AMTS greater than or equal to 7 (25.53% vs. 17.24%; p > 0.05).

One-hundred twenty seven out of 157 (80.89%) of the patients were from their own home; 30/157 (19.11%) of the patients were from a residential or care home (80.89% vs. 19.11%; p < 0.00001). Eighty-seven out of 157 (55.41%) were treated with either HH or total hip replacement (THR), 30/157 (19.10%) were treated with an IMN, and 40/157 (25.48%) were treated with a DHS.

The average preoperative Hb (measured in g/L) was 123.01g/L ±2.701 (±2.2%) (120.309-125.711; 95% CI). Ninety-eight out of 157 (62.42%) patients had either two or more comorbidities, while 59/157 (37.58%) had fewer than two comorbidities. 

Ten out of 157 patients had died within 30 days of presentation (6.37%); 13/157 patients (8.28%) had died within 60 days of presentation. Twenty-three out of 157 (14.65%) had died within six months, and 32/157 (20.38%) had passed away at 12 months. The mean NHFS was 5.0 - representing a 30-day mortality risk prediction of 5.99%. The mean SORT 30-day mortality was 8.28%. The actual mortality rates and the predicted models are listed in Table 2.

**Table 2:** Thirty-day, 60-day, six-month, and 12-month actual mortality rates. The NHFS and SORT specifically predict only the 30-day mortality. The NHFS value was significantly more accurate than the SORT score (p < 0.05). NHFS: Nottingham Hip Fracture Score, SORT: Surgical Outcome Risk Tool

	Number (N = 157)	Percentage (%)	Predicted NHFS 30-day mortality	Predicted SORT 30-day mortality
Actual 30-day mortality	10.00	6.37	5.99	8.28
Actual 60-day mortality	13.00	8.28	-	-
Actual six-month mortality	23.00	14.65	-	-
Actual 12-month mortality	32.00	20.38	-	-

The H-L test p-value for the NHFS was 0.84, suggesting a model of good fit. For the SORT score, the H-L test, even though the SORT model overpredicts mortality slightly (Observed/Expected O/E = 0.77), the p-value was 0.39. There was no evidence of poor fit for either mortality prediction tool; however, the NHFS appears to have a better fit to the observed rate.

The mean NHFS was 5.0 - representing a 30-day mortality risk prediction of 5.99%. The mean SORT 30-day mortality was 8.28%. When compared to the actual 30-day mortality of 6.67%, the NHFS was not significantly different to this value (5.99% vs. 6.67%; p = 0.453; p > 0.05); however, the SORT 30-day prediction of 8.28% was significantly higher than the actual mortality rate (8.28% vs. 5.99%; p < 0.00001); therefore, the NHFS was significantly more accurate than the SORT score in predicting 30-day mortality for this particular cohort over this particular time period. The 60-day mortality in this cohort was 13/157 (8.28%), the six-month mortality was 23/157 (14.65%), and the 12-month mortality was 32/157 (20.38%).

**Figure 4:**
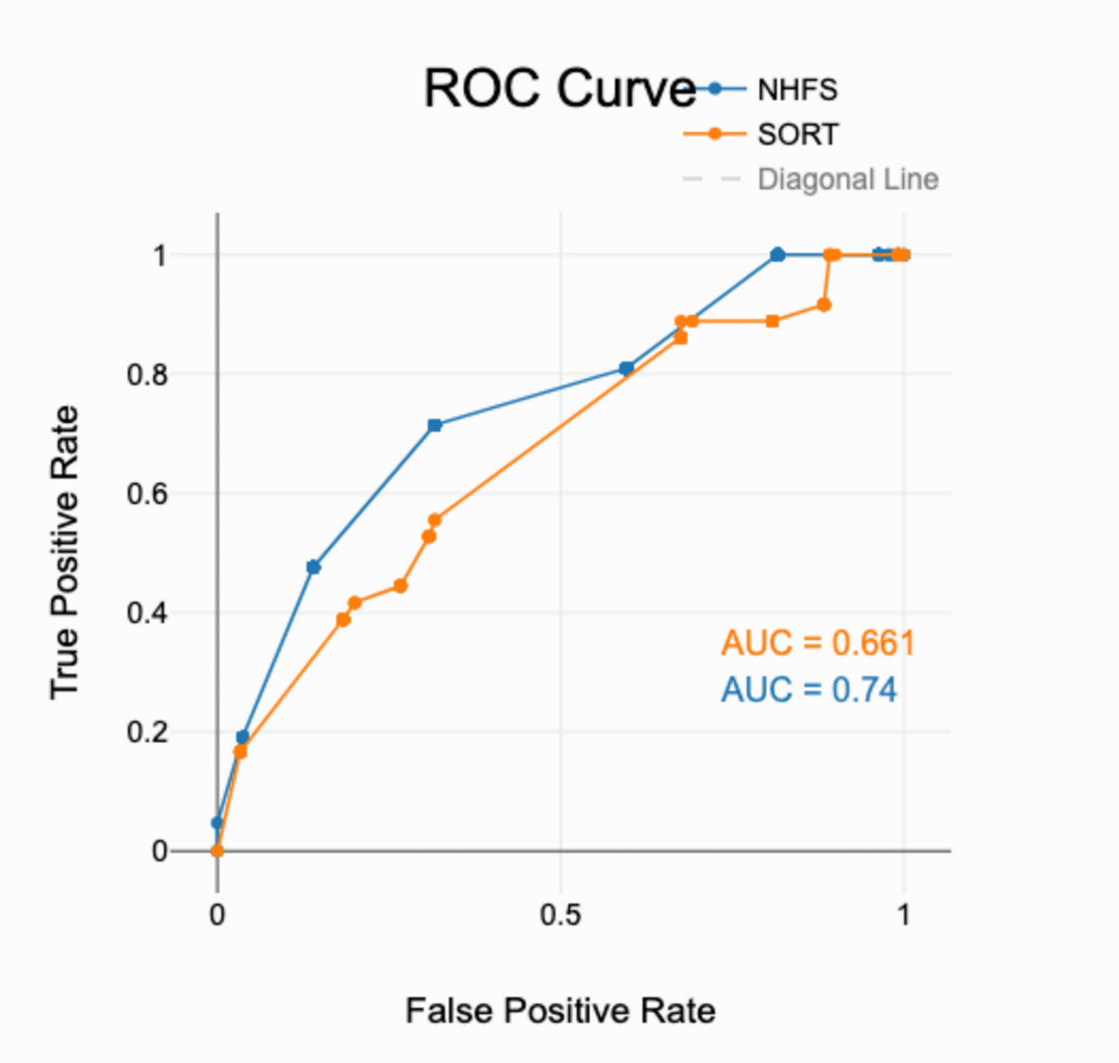
Receiver operator curve for the NHFS and SORT showing the false-positive and true-positive rates. The area under the ROC curve (AUC) for the Nottingham Hip Fracture Score (NHFS) curve is 0.74. The area under the ROC curve (AUC) for the Surgical Outcome Risk Tool (SORT) curve is 0.661. DeLong's test for two independent ROC curves showed p < 0.05.

Figure 4 shows the receiver operating characteristic (ROC) curve for the NHFS and SORT. For the NHFS, the area under the ROC curve (AUC) was 0.74, 95% CI (0.62, 0.86), SE = 0.06, p < .001. This indicates that the NHFS significantly discriminates between the outcome groups, and its performance is statistically better than chance. For the SORT, the area under the ROC curve (AUC) was 0.66, 95% CI (0.56, 0.76), SE = 0.05, p = 0.002. This indicates that the SORT significantly discriminates between the outcome groups, and its performance is statistically better than chance. 

The NHFS is separated into each of the nine possible scores, and the predicted versus actual 30-day mortality is displayed in Table 3. The mean NHFS was 5.0 - representing a 30-day mortality risk prediction of 5.99%. The mean SORT 30-day mortality was 8.28%. When compared to the actual 30-day mortality of 6.67%, the NHFS was not significantly different to this value (5.99% vs. 6.67%; p = 0.453; p > 0.05); however, the SORT 30-day prediction of 8.28% was significantly higher than the actual mortality rate (8.28% vs. 5.99%; p < 0.00001); therefore, the NHFS was significantly more accurate than the SORT score in predicting 30-day mortality for this particular cohort over this particular time period. The 60-day mortality in this cohort was 13/157 (8.28%), the six-month mortality was 23/157 (14.65%), and the 12-month mortality was 32/157 (20.38%). 

**Table 3:** Separations of the NHFS, number of patients' distribution, and the comparison between the predicted 30-day mortality and the actual 30-day mortality. NHFS: Nottingham Hip Fracture Score

NHFS	Number of patients (N = 157)	Predicted 30-day mortality (%)	Actual 30-day mortality (%)
0	2	0.4	0
1	1	0.6	0
2	2	1	0
3	20	1.7	0
4	34	2.8	2.94
5	40	4.6	0
6	29	7.4	17.24
7	20	11.8	10
8	8	18.2	25
9	1	27	0

## Discussion

The results of our paper inspire multiple discussion points. In this study, it is evident that the NHFS appears to be a more accurate predictor of 30-day mortality than the SORT in this particular hospital setting. The NHFS is simpler to utilise in the experience of the authors during the initial work-up for the admission of the patients. The ROC curve in Figure 4, calculated for each risk stratification score, indicates that, in this hospital setting, the NHFS and SORT were both valid prediction models. The AUC of the NHFS was greater than that of the SORT (0.74 vs. 0.66; p < 0.05), suggesting that the NHFS was a significantly better fit for true mortality. One reason for this is that the SORT does not take into account the important variables, as we shall explore in this paper, that contribute explicitly to hip fracture mortality. It does not take into account the sex of the patient, the preoperative Hb, nor the mental state or the living arrangement of the patient. These variables have been explored in existing literature and are thought to each contribute significantly to NOFF outcomes.

Looking at the demographics of patients included in this retrospective study, the cohort was made up of 106 women and 51 men (106/ 157 women to 51/157 men). This was a statistically significant difference in sex differences within the cohort (67.52% women vs. 32.48% men; p < 0.00001). It is known that osteoporosis and decreased bone mineral density largely contribute to the prevalence of NOFFs, of which women are more prone. These are attributed to the differences in Testosterone, decreased availability of bone protective properties of oestrogen in post-menopausal women and increased age due to increased life expectancy of women compared to men. To explore this finding further, we searched existing literature. Alpantaki et al. [8] conducted a nine-year retrospective cohort study of 2,430 patients aged over 65 with NOFFs that were surgically treated. They found that women suffered hip fractures 2.9 times more often than men. Among women aged 50-54 years, the relative risk (RR) of hip fracture is significantly higher in postmenopausal women compared to premenopausal women [9]. Within the postmenopausal group, hip fracture incidence rose sharply with age (p < 0.001), with rates approximately seven times higher at ages 70-74 than at 50-54 years. 

Early recognition of cohorts with high mortality risk allows early optimisation through the utilisation of blood transfusion, nutritional optimisation, early administration of analgesia through fascia iliaca blocks [10], and intravenous fluid resuscitation. Postoperatively, these patients can be targeted with early mobilisation, chest physiotherapy, and orthogeriatric review [11] to enhance recovery. One hundred twenty-seven out of 157 (80.89%) patients were from their own home; significantly, more than 30/157 (19.11%) patients were from a residential or care home (80.89% vs. 19.11%; p < 0.00001). The residence of patients is included in the NHFS as a prognostic indicator. This is likely because patients with decreased independence or higher frailty scores are more likely to need the support provided by Care Homes, which is largely reflected in the mobility status. The ability to physically navigate their own homes, use stairs, and cognitive ability to organise day-to-day living likely reflects a higher functionality baseline compared to the patients who require increased support [12,13]. 

Preoperative Hb is also utilised as part of the NHFS. All preoperative Hb levels were collected 24 hours before operative management to allow for valid comparisons to be drawn. Understandably, preoperative Hb levels are linked closely with the postoperative Hb levels as blood loss tends to be equivocal across the surgical options. Hb level can determine the risk of anaemia, weakness, hypotension, type 2 myocardial Infarction risk, risk of needing blood transfusions, ability to work with physiotherapy, and length of hospital stay. Haddad et al. conducted a multivariate analysis of 626 patients [14]; the mean age was 76.27 ± 9.57 years (81.696-84.304; 95% CI). Three- and six-month mortality rates were 11.2% and 14.1%, respectively. The highest mortality was observed in patients aged over 80 years and in male patients. The Hb level upon admission was lower in individuals who died within six months compared to those who survived (109.7 g/L ± 2.02 vs. 11.99 ± 2.39, p < 0.001), and in the multivariate analysis was actually an independent risk factor for postoperative mortality. 

Doherty et al. [15] conducted a retrospective study of over 3,000 NOFF patients assessing the mortality-predicting ability of the NHFS and AMTS. For mortality, they found that the predictive ability (C-statistics) for the NHFS was similar to using ASA and AMTS alone. The AMTS, therefore, plays a crucial role in identifying vulnerable patients and is relatively easy to perform and document. In this paper, we found that more patients with a lower AMTS were at an increased risk of 12-month mortality than those with scores over 7; however, this was not statistically significant. Forty-one out of 157 (26.11%) had an AMTS score of under 7, and 116/157 (73.89%) of patients had a score of 7 or more on the AMTS. Twelve out of 41 (25.53%) of those with AMTS less than 7 died within 12 months, and 20/116 (17.24%) with AMTS greater than or equal to 7 (25.53% vs. 17.24%; p > 0.05). 

In this paper, those with an NHFS of 5 or below had a 30-day mortality rate of 1/99 (1.01%), and those with an NHFS over 5 had a 30-day mortality rate of 9/58 (15.52%). The SORT score of 30-day predicted mortality was rather high at 8.28% for 30-day mortality. The SORT score factors in the operative fixation choice. This paper did not look at outcomes associated with surgical choice; however, analysing existing literature, there are robust studies looking at fixation methods. Czerwonka et al. analysed 29,809 Intertrochanteric NOFFs [15] and compared the outcomes associated with HH, THR, and IMNs. Primary HH is associated with an increased 30-day reoperation rate and decreased need for blood transfusion, but there were no other significant differences in postoperative morbidity identified among IMN, THR, and HH in the treatment of IT fractures.

Deng et al. [16] conducted a study of 251 NOFF patients who either underwent a HH or IMN for fixation or Intertrochanteric NOFFs. Statistically significant differences were not observed in the clinical outcomes, mobility, overall survival, or all-cause mortality after surgical treatment between the groups receiving IMNs and the HH group (p > 0.05). Nevertheless, among patients aged ≥85 years, the IMN group demonstrated a lower rate of all-cause mortality than the HH group did (p < 0.05). One explanation for the differences in the elderly population may be explained by the longer anaesthetic time for HH, especially those with cemented stems. The wound size for DHS and IMNs remains smaller in length on average than those who undergo a HH, especially with a posterolateral approach. The smaller wound hypothetically would require less physiological demand to heal, a smaller area for infections to occur and for those with cognitive impairment, less surface area to interfere with. The longer anaesthetic time and larger wound may also result in increased amounts of postoperative analgesia, commonly in the form of opioids [17], thereby increasing the occurrence of adverse reactions, poor mobility, constipation, decreased rates of successful trials without catheters (TWOCs) and overall longer stays in the hospital [18]. 

The findings of this paper were corroborated by Tang et al. [19], who analysed 212 patients with NOFFs. They compared the accuracy of the NHFS versus the SORT score of 30-day mortality and found, similar to this paper, that the NHFS was more accurate (p < 0.05). They hypothesised that this was because the NHFS was developed as a specific scoring tool for NOFFs, whereas the SORT was created as a generic tool for surgical outcomes. This paper also separated the NHFS scores into individual scores; however, the prediction accuracy was not present across all the scores. This finding is mirrored in the paper by Tang et al., who found that the overall mortality prediction rate by the NHFS was not accurate on all levels (0 to 9). However, some of the NHFS had small sample sizes; hence, the validity of assessing accuracy at each level may be somewhat of a limiting factor. 

One limiting factor of the NHFS is that there is no clear definition of comorbidities. Whilst, in this study, this was defined as patients with cardiovascular dysfunction, diabetes, hypertension, malignancies (excluding benign skin lesions), osteoporosis, respiratory disease, cognitive impairment, renal and gastrointestinal pathology, some clinicians may include comorbidities that were less severe, such as poor hearing and visual impairment, such as cataracts, and mild osteoarthritis, given that these factors also have a large influence on patient outcome; it would be prudent to perhaps include these as part of future studies involving hip fracture comorbidities [20]. This inter-clinician variance in how the NHFS is used suggests that when assessing the literature, it should be made clear the criteria for which comorbidities were chosen. It is recognised that SORT overprediction may reflect the small sample variability, not only the scoring design. One limitation of the SORT is that the surgical procedure in the list of operations to choose from did not always accurately reflect the operation performed for the patient; rather, there was a choice of the closest match. The advantage the SORT has over the NHFS is that there is no requirement for blood tests to calculate the SORT score; therefore, patients with difficult venous access can still have a predictor model at the clinician’s disposal without the preoperative Hb. 

As a tertiary outcome, we aimed to explore the quality of the clerking process for patients and put forth suggestions for improvements. Interestingly, out of the 78 patients who were excluded due to insufficient data, all of them were excluded due to a lack of documentation regarding the preoperative AMTS. This highlights an important point regarding thorough work-up and clerking of patients presenting with NOFFs. The presence of the AMTS within the NHFS may promote an increased rate of cognitive impairment assessments if the NHFS utilisation becomes standardised practice within this particular hospital. The reasons for missing AMTS scores from documentation are not entirely clear. It may be perhaps the clinician did not deem the AMTS score either necessary, as the patient was so clearly orientated to time, place and circumstance or because the patient had long-standing cognitive impairment that the AMTS was presumed to be 0, but not officially recorded. This does offer a discussion point for improvement in this particular hospital as a quality improvement project. One suggestion is to integrate the AMTS into CPD, such that before a patient has been admitted under the Orthopaedic Speciality, their AMTS must be calculated. The other option is to offer financial incentives to trusts that upload the AMTS to the National Hip Fracture Database and audit the information being presented. These patients normally first present to the Accident and Emergency department via ambulance, before being admitted under Orthopaedics, and they commonly receive the fascia iliaca block. Before the block is done, the Emergency Medicine physician could calculate the AMTS as part of the consenting process.

Study strengths and limitations

The strengths of this study include the NHFS component breakdown to assess each variable separately. This allows us to compare and contrast the effect that each variable has on patient mortality in great detail through the use of existing literature in addition to the findings of this paper. We also ensured that the data collected for each patient was accurate. Each clerk's notes were checked and corroborated by a consultant orthopaedic surgeon during the post-take ward rounds, whereby the accuracy of the admission information was checked by the senior clinician, and any changes to the clerking were made before the data was collected. This ensured that any errors or misjudgements in each data point collected were nullified before analysis.

Whilst this paper contributes to the wealth of data on hip fractures as an exploratory study and adds value as additional evidence base, the main limitation of this study is the relatively small sample size of 157 patients. Ideally, there would have been over 225 patients, but due to a lack of proper documentation, it was not possible to accurately calculate the AMTS and subsequently the NHFS for the patients excluded from the study. The exclusion of patients with AMTS may lead to selection bias. The patients who had no formal AMTS calculated and documented were likely those with either severe cognitive impairment, such as advanced dementia or acute confusion, that which calculating and documenting the mental state was seen as unnecessary by the admitting clinician. This would be a selection bias to not include these patients in the study, as patients with low AMTS would be likely to have higher mortality rates, and this may have skewed the data in favour of patients with relatively favourable outcomes. A clinical improvement project by Hao et al [21] examined this very issue in their own clinical setting. They found importance in educating clinicians on the importance of the AMTS and the rationale for the calculation, either via posters or by identifying ways to make AMTS calculation simpler. The AMTS assessment could be part of clerking, similar to how Venous Thromboembolic risk factors get flagged up on the CPD system each time a patient’s record is accessed. The small sample size of the study results in anomalous results of the patient skewing the results with an increased impact on the ROC curve, whereas higher-powered studies would provide a more representative AUC. The other limitation of the paper is that there were no additional criteria for patients who had been delayed beyond the recommended 36-hour timeframe from admission to operative fixation. The delayed treatment would have likely contributed to increased actual mortality; however, neither the NHFS nor the SORT incorporate the BPT into their calculations. The final limitation of the study is that the NHFSs were not segregated according to the operative fixation utilised to nullify the variability of surgical fixation. Future studies would therefore integrate Multiple Regression models to analyse a variety of factors in the mortality rate of NOFF patients.

## Conclusions

This paper suggests there is perhaps greater utility in predicting 30-day mortality through the use of the NHFS over the SORT; however, one must be cautious in determining clear superiority. There remains the need to conduct large studies across multiple centres for high-powered studies to compare the scoring systems, in addition to the use of multivariate analysis to negotiate the impact of individual variables. The main rationale for this finding is that a dedicated tool, which incorporates measures relevant to the cohort most affected (AMTS in the elderly) and measures relevant to the management (Hb), affects the accuracy. Much work is yet to be done in fine-tuning predictor tools; however, it is elucidated that ensuring thorough clerking allows us to identify vulnerable patients early and optimise this cohort to allow decreased mortality, decreased length of stay, and improved mobility, thereby increasing the good quality of life years gained for patients.
